# Asymmetrical Parental Investment Across Breeding Stages in the Globally Endangered Egyptian Vulture (*Neophron percnopterus*) in Nepal

**DOI:** 10.1002/ece3.74406

**Published:** 2026-10-11

**Authors:** Milan Baral, Anisha Neupane, Sandesh Gurung, Tulsi Ram Subedi

**Affiliations:** ^1^ Nepalese Ornithological Union Kathmandu Nepal; ^2^ Department of Biological Sciences University of Lethbridge Lethbridge Alberta Canada; ^3^ Himalayan Raptors Bhaktapur Nepal

**Keywords:** aggression, bi‐parental care, breeding ecology, food provisioning, scavenger, South Asia

## Abstract

Understanding sex‐specific parental roles is critical for interpreting reproductive strategies in long‐lived, monogamous birds with low fecundity. Birds of prey often exhibit asymmetric biparental care; however, empirical evidence from South Asia, mainly on *Neophron percnopterus ginginianus* sub‐species remains limited. We investigated breeding phenology and sex‐specific parental investment between 2021 and 2024, across breeding stages of the globally endangered Egyptian Vulture, *Neophron percnopterus ginginianus* , in the Kaski district, which supports a high density of the species' breeding population. We monitored 46 breeding attempts to document breeding phenology and conducted behavioral observations at 38 unique breeding attempts from 17 nests, comprising 20 nest‐building, 18 incubation, and 18 chick‐rearing attempts. Using generalized linear mixed‐effects models and an information‐theoretic model‐selection approach, we evaluated sex‐specific parental investment and the influence of offspring development and environmental conditions on breeding behavior. Breeding onset varied among nests and years, with nest‐building occurring predominantly in March, and the overall breeding cycle lasted approximately 18.4 weeks. Egyptian Vultures exhibited bi‐parental care throughout the breeding cycle, but parental roles were asymmetric and varied among stages. Males delivered nesting materials more frequently than females, whereas females contributed more to nesting material maintenance. During incubation, both sexes contributed similarly to incubation effort, while incubation bout duration decreased with increasing sunlight exposure. During chick‐rearing, females were most likely to attend and spent longer duration at nests and feeding chicks when present, whereas males delivered more food more frequently. Food provisioning rate increased with chick age, whereas feeding duration decreased with increasing precipitation. Chick became increasingly independent as they developed, with the probability of self‐feeding increasing with chick age. Aggression by chicks toward parents also increased with chick age and was directed predominantly toward females. Overall, our findings reveal complementary and stage‐dependent parental roles in Egyptian Vultures, with males contributing more strongly to resource acquisition and females investing more in direct offspring care. These findings provide detailed evidence of how parental roles shift across the breeding cycle and offer region‐specific insights into the reproductive ecology of this globally endangered scavenger.

## Graphical abstract

Both mates of an Egyptian Vulture pair work together to raise chicks, but their roles differ. Males mostly bring food and nesting material, while females spend more time at the nest caring for eggs and chicks. Parental roles change over time; during incubation, males and females share duties more equally. As chicks grow, females focus more on feeding and guarding them, while males increase food delivery. Chick behavior changes with age; older chicks become more independent—they feed themselves more and show more aggressive behavior, especially toward females.

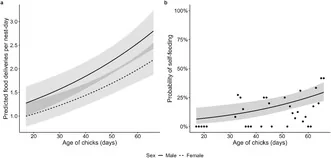

## 1 Introduction

Parental care is one of the most variable social behaviors and provides an excellent model for understanding cooperation between breeding partners (Remeš et al. 2015). Although parental care increases offspring survival, it imposes energetic and opportunity costs on parents through reduced time available for self‐maintenance and other activities. In biparental species, these costs need not be equally distributed between pair members because males and females can specialize in different components of care. Such task specialization is particularly evident in birds of prey, in which females usually undertake incubation, brooding, and nestling provisioning, while males contribute disproportionately to food acquisition and provisioning (Eldegard et al. 2003; Newton 2010; Eldegard and Sonerud 2012; Morant Etxebarria et al. 2019). Sex‐specific specialization in complementary parental tasks may facilitate efficient biparental care, particularly when offspring require multiple forms of care simultaneously (Henshaw et al. 2019).

Large raptors provide a useful system for investigating how parental responsibilities are divided between pair members because they are generally long‐lived, socially monogamous, and characterized by low fecundity and prolonged offspring development (Morant Etxebarria et al. 2019). Biparental care in such species includes nest construction and maintenance, incubation, egg turning, nest attendance, food provisioning, and direct offspring care, and the relative contribution of each parent may differ among these activities and across breeding stages (Morant Etxebarria et al. 2019). Importantly, parental investment is not fixed but may vary with offspring requirements and environmental conditions. Chick development can alter the demand for attendance and provisioning, while temperature, precipitation, and solar exposure can influence the energetic and temporal cost of incubation, foraging, and nest attendance (Cresswell et al. 2003; Kovařík et al. 2009; Smith et al. 2012; Bulla et al. 2016; Zuberogoitia et al. 2018). Consequently, understanding parental care requires consideration not only of the amount of care provided by each sex, but also of how pair members divide different parental tasks and how these contributions change with breeding stage, offspring development, and environmental conditions.

Vultures are particularly suitable for examining such division of parental roles because they are long‐lived, monomorphic, socially monogamous birds that maintain stable pair bonds and exhibit prolonged biparental care (Newton 2010). Their peculiar foraging ecology, which involves exploiting spatially and temporally unpredictable food resources, often requires prolonged absences from the nest by one or both parents, thereby affecting patterns of nest attendance and parental investment (Jackson et al. 2008; Deygout et al. 2010). Understanding these behaviors is also relevant to conservation because changes in food availability, disturbance, or environmental conditions could alter the ability of breeding pairs to allocate sufficient care to offspring (Brooker et al. 2016; Merrick and Koprowski 2017). Previous studies of Egyptian Vultures ( *Neophron percnopterus* ) indicate biparental care with sex‐specific task specialization. In European populations, females contributed more to incubation, brooding, and egg turning, whereas males contributed more to nest‐material delivery (Morant Etxebarria et al. 2019; Yordanov et al. 2022). However, despite being identified as a high‐priority raptor species for conservation in the world (McClure 2025), detailed information on parental behavior remains limited across much of the species' South Asian range, mainly in *N.p. ginginianus* (but see Kumar et al. (2020)).

In Nepal, Egyptian Vultures are resident breeders, mainly distributed from central to western Nepal (Inskipp et al. 2016), with particularly higher abundance and nesting probabilities in the mid‐hills of Kaski District (Gurung et al. 2023; Baral, Neupane, and Subedi 2026). Existing research in Nepal has focused predominantly on population (Ghimire et al. 2020; Baral, Neupane, and Subedi 2026), habitat (Khim Bahadur et al. 2019; Gurung et al. 2023; Baral, Neupane, and Subedi 2026), feeding (Baral, Neupane, Gurung, et al. 2026), and breeding success (Subedi and DeCandido 2015), leaving breeding behavior and sex‐specific parental roles largely unexplored.

Current knowledge on parental care in Egyptian Vultures is derived from populations outside Nepal, particularly Europe (Donazar et al. 1994; Morant Etxebarria et al. 2019) and, to a lesser extent, India (Kumar et al. 2020). However, Egyptian Vultures breeding in Nepal experience ecological conditions that differ substantially from those of well‐studied European populations. In the Pokhara region of Kaski District, breeding occurs in a humid subtropical, strongly monsoonal environment, where annual precipitation can approach 4000 mm and more than 80% of rainfall occurs during the summer monsoon (May–July) with mean summer rainfall of 800 mm (Paudel 2020; Fischer et al. 2022). In contrast, the northern Spanish population studied by Morant Etxebarria et al. (2019) experiences a wet Atlantic climate with approximately 1200–2000 mm of annual precipitation. In addition, Egyptian Vultures in Nepal are resident breeders and frequently exploit food resources associated with human settlements, slaughterhouses, dumping sites, and livestock, whereas European populations are migratory and occur within different livestock‐management and supplementary‐feeding systems (Milchev et al. 2012; Mateo‐Tomás and Olea 2015; Baral, Neupane, and Subedi 2026). These differences in climatic seasonality, annual‐cycle strategy, and food‐resource landscapes may alter the energetic and temporal constraints associated with incubation, nest attendance, foraging, and food provisioning. Consequently, evaluating parental role under Nepalese conditions is important for determining whether patterns documented elsewhere in the species' range are generalizable to South Asian populations and for developing locally appropriate conservation strategies.

In this study, we examined breeding phenology and sex‐specific parental investment in Egyptian Vultures in the Kaski District, Nepal. Specifically, we aimed to (a) characterize breeding phenology; (b) quantify the relative contributions of males and females to nest building, incubation, and chick rearing; and (c) determine whether parental behaviors varied with offspring developmental stage and environmental conditions. Based on previously documented division of parental roles in raptors and Egyptian Vultures, we predicted that parental investment would be task‐ and stage‐specific rather than uniformly distributed between pair members. Specifically, we expected males to contribute more to resource‐acquisition activities, such as nest‐material and food delivery, whereas females would contribute more to direct offspring‐care activities, including incubation, nest attendance, and chick feeding. We further predicted that parental behavior would vary with offspring development and environmental conditions because the requirements and costs of parental care change throughout the breeding cycle.

## 2 Methods

### 2.1 Study Area

We conducted our study in the Kaski district, located in the mid‐hills of central‐western Nepal (Figure 1). This region has a subtropical climate, with mean monthly temperatures ranging from 6.8°C in January to 20.9°C in April, and average annual precipitation of 238.2 mm. The elevation ranges from 450 m to 7969 m above sea level, with a predominantly steep, rugged landscape characterized by rocky cliffs interspersed with forests (~45%), agricultural lands, human settlements, and natural water bodies. Several major rivers, including the Kali, Seti, Yemdi, Gandaki, and Mardi, pass through the district, forming wide gorges with high cliffs that provide suitable breeding habitats for the Egyptian Vultures (Gurung et al. 2023).

**FIGURE 1:**
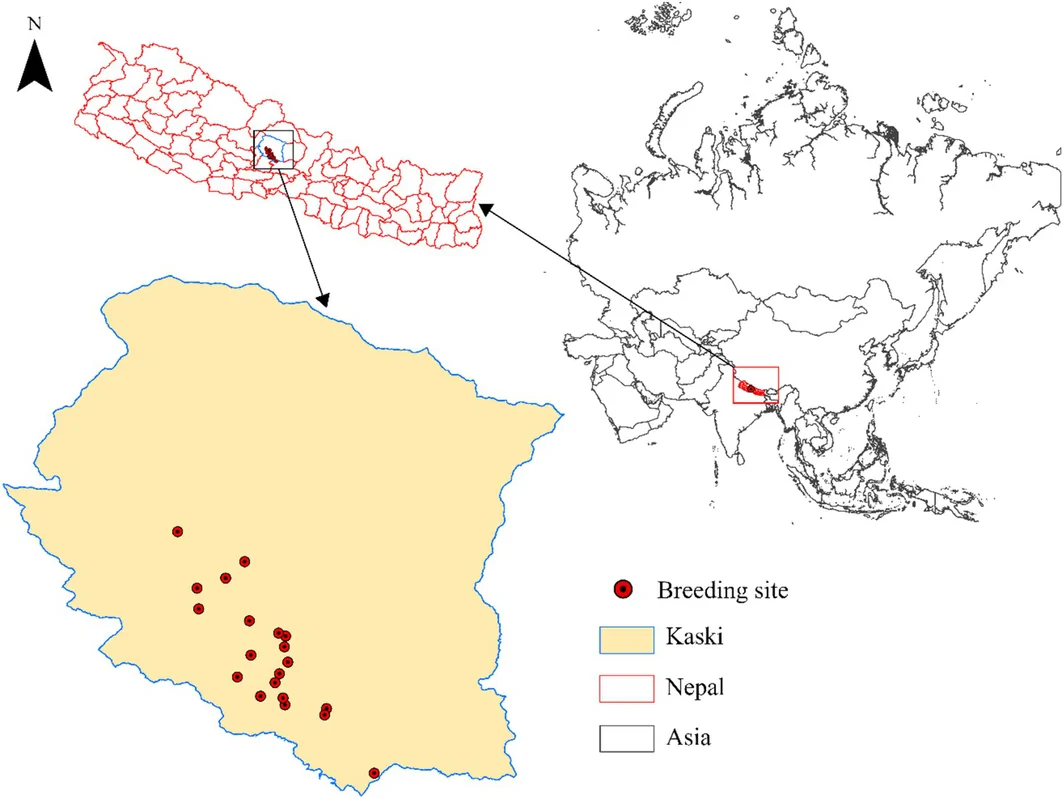
Map showing the location of breeding sites within the Kaski District of central‐west Nepal. Red dots indicate surveyed breeding sites. The shaded polygon represents the boundary of the Kaski District, while the red outline indicates Nepal's national boundary and black outline represents Asia.

Kaski district harbors all nine species of vultures recorded in the Indian subcontinent (Inskipp et al. 2016; Ghimire et al. 2019) and hosts almost 50% of the national population of the Egyptian Vulture (Baral, Neupane, and Subedi 2026). Likewise, the breeding density recorded in this area is higher than that recorded in any other area in the country (Gurung et al. 2023). So, we used these breeding populations as a significant tool to understand the breeding phenology and parental care roles of the Egyptian Vulture in Nepal.

### 2.2 Nest Site Survey

Between 2019 and 2024, we surveyed known (Gurung et al. 2023) and potential breeding territories of the Egyptian Vulture in the Kaski district, Nepal, and detected 20 active cliff nesting territories in the study area. Given that the breeding phenology of vultures may vary with food availability across seasons and geographic locations (Newton 2010; Renner and Zohner 2018), we conducted multiple surveys between February and March in each study year to identify active nests. We thoroughly inspected potential caves, ledges, and cavities in cliffs for evidence of nesting. To locate tree‐nests, we systematically searched suitable trees within areas of Egyptian Vulture activity, visually inspecting the canopy, major branches, and branch forks for nests and nesting materials. We considered a nest or territory as active if we observed breeding activities, including nest building or incubation (Margalida et al. 2012; Oppel et al. 2017). However, if we detected only courtship displays, copulation, or territorial defense, we monitored sites for at least 4 h to confirm breeding activity.

### 2.3 Breeding Phenology

Since 2021, we monitored 53 breeding attempts (7 in 2021, 12 in 2022, 17 in 2023, and 17 in 2024) of Egyptian Vultures to study the species' breeding phenology. We visited each active nest multiple times throughout the breeding season, with visits concentrated during February/March, March/April, May/June, and July to determine the timing of major breeding events. Breeding stages were identified based on direct observations of nesting behavior and nest contents, following the general breeding sequence described for Egyptian Vulture in India (Kumar et al. 2020).

We classified the breeding cycle into nest building, pre‐laying, incubation, chick‐rearing, and fledging stages. We identified nest building was identified by repeated material delivery and arrangement of nesting materials by adults. Following nest completion, adults were occasionally observed adopting an incubation‐like posture before the first egg was laid; this period was classified as the pre‐laying stage. During focal observations, we confirmed the presence or absence of eggs when attending adults stood, repositioned, or left the nest, allowing us to observe the nest contents. After egg laying, parental changeovers provided additional opportunities to confirm the presence of eggs. We therefore classified incubation‐like sitting when no egg was present as pre‐laying behavior, and considered incubation to have commenced only after confirming the presence of an egg. Chick rearing began following hatching and continued until fledging, which was determined when the nestling left the nest.

For each breeding attempt, we recorded the dates of major phenological events and calculated the duration of each breeding stage where both the beginning and end of the stage could be determined. We then summarized the timing and duration of breeding stages across breeding attempts and years.

### 2.4 Breeding Behavior

Between 2021 and 2024, we examined the breeding behavior of Egyptian Vultures at 17 unique active nests, incorporating 20 nest‐building attempts, 18 incubation attempts, and 18 chick‐rearing attempts. We observed each nest for at least 5 days per breeding stage (nest‐building, incubation, and chick‐rearing), from 6 am to 6 pm. We didn't include the fledgling stage in the behavioral study, as it was difficult to monitor the behavior of fledglings away from the nests.

We differentiated male and female Egyptian Vultures using a combination of sexually dimorphic and behavioral characteristics. Males generally exhibited deeper orange‐yellow facial skin, whereas females showed a more yellow coloration (Clark and Schmitt 1998; Kumar et al. 2020). We also used the prominence of the dark smudge below the eye as supplementary field characteristics, as reported in India (Kumar et al. 2020), while recognizing that this characteristic has not been established as a universally reliable sexing criterion across the species' range. Differences in brood‐patch development during the early breeding period provided additional supporting characteristics (Kumar et al. 2020). Sex assignments of pair members were further verified through direct observations of copulation. The breeding Egyptian Vultures monitored in our study were considered to belong to the Indian subspecies *Neophron percnopterus ginginianus* , which is the subspecies occurring in Nepal and most of the Indian subcontinent (Mishra et al. 2018).

All observations were conducted from approximately 100 m away from the nests, using camouflage and natural hides to minimize disturbance to breeding birds. We recorded behavioral data from 6 am to 6 pm using binoculars (Celestron 10 × 42), a spotting scope (Vortex 20–60 × 80, angled), and cameras (Nikon Coolpix P1000 and Canon EOS 7D Mark II). We obtained daily temperature and precipitation data (~9 km resolution) for each nest location from the ERA5‐Land Daily Aggregated dataset (ECMWF/ERA5_LAND/DAILY_AGGR) using Google Earth Engine (Gorelick et al. 2017; Munoz‐Sabater et al. 2021).

#### 2.4.1 Copulation and Nest Defense

We recorded copulation attempts of Egyptian Vultures during the nest‐building stage. Each recorded copulation attempt was treated as one event. We summarized the total number of copulations, the number of territories and breeding attempts, copulation duration, and copulation substrates. Because copulation durations were non‐randomly distributed and sample sizes differed among substrates, we compared duration among cliff, tree, and nest substrates using a Kruskal–Wallis test. When the overall test was significant, pairwise Wilcoxon rank‐sum tests were conducted with Benjamini–Hochberg adjustment for multiple comparisons.

We recorded the nest defense behavior throughout the breeding cycle during focal observations of breeding pairs. A defense event was defined as any vocal (hoarse hissing) or physical (chasing) response by an adult Egyptian Vulture toward a potential nest intruder. During each observation session, all nest defense events were recorded, together with the identity of the defending parent (male or female), the breeding stage, and the type of intruder.

#### 2.4.2 Nesting Material and Nest Building

During the nest‐building stage (February–March), we recorded the types and colors of nesting materials, the timing of material delivery, sex‐specific roles, and copulation attempts. We calculated diversity indices (Shannon and Simpson) and the richness of nesting material type and color. We analyzed the frequencies of material delivery and material management using generalized linear mixed models (GLMMs) in R using the *glmmTMB* package (Magnusson et al. 2017) with a negative binomial error distribution. For each behavior, the response variable was the number of events performed by each sex per observation day. Sex and year were included as fixed effects, and nest‐day identity was fitted as a random intercept to account for paired male and female observations within the same observation day.

We used Pearson's Chi‐squared test to understand how males and females differ in material types (overall material and anthropogenic vs. natural origin) and colors delivered to the nest as well as the association between the sex delivering nesting material and the sex managing it afterwards. All model assumptions, including overdispersion and residual patterns, were checked using the DHARMa packages in R (Hartig and Hartig 2017).

### 2.5 Incubation

For each active nest, we recorded the incubation behavior from focal observations conducted throughout the incubation period. Individual incubation bouts were assigned to the attending parent and aggregated by breeding attempt, observation day (nest‐day), and parental sex. For each sex on each nest‐day, we calculated total daily incubation duration, incubation bout frequency, mean incubation bout duration, egg‐turning frequency, nest‐repair frequency, sunlight duration, and anthropogenic disturbances. Because disturbances occured only in a few nests, we excluded them from our analyses. Incubation bouts were also arranged sequentially to identify changeovers between parents. We defined changeover as an incubation bout immediately followed by a bout performance by the opposite sex, whereas consecutive bouts by the same parent were classified as same‐sex continuations.

Sex differences in daily incubation duration were analyzed using a Gaussian generalized linear mixed model (GLMM), whereas incubation bout duration was analyzed using a Gamma GLMM with a log link. Egg‐turning and nest‐repairing frequencies were analyzed using a Poisson GLMM (Magnusson et al. 2017), and changeover occurrence was analyzed using a binomial GLMM. All models were fitted using the *glmmTMB* package in R (Magnusson et al. 2017).

To evaluate influence of environmental factors on incubation behavior, we developed separate biologically motivated candidate models by adding one predictor at a time (age of eggs, sunlight duration, daily temperature, or daily precipitation) to the baseline model containing sex and year. We standardized continuous predictors before analysis, ranked candidate models using Akaike's Information Criterion corrected for small sample size (AICc), and interpreted the top‐ranked model.

### 2.6 Chick‐Rearing

During chick‐rearing, for each active nest, we recorded parental and chick behaviors from focal observations. We aggregated behavioral observations by breeding attempt, observation day (nest‐day), and parental sex where appropriate. For each sex on each nest‐day, we calculated parental nest‐attendance duration, feeding duration, and food provisioning frequency. Food‐provisioning events were assigned to the parent that delivered the food, whereas events involving stored food or an unidentified delivering parent were excluded from sex‐specific provisioning analyses. We also recorded self‐feeding by chicks, use of previously stored food, and chick aggression during feeding.

We analyzed parental nest attendance separately as attendance occurrence and attendance duration. We analyzed attendance occurrence using a binomial generalized linear mixed model (GLMM), whereas we used a Gamma GLMM with a log link to analyze attendance duration, conditional on attendance occurring (> 0 min). We summarized feeding occurrence descriptively because females fed chicks on every monitored nest‐day, resulting in complete separation that precluded binomial modeling. We used a Gamma GLMM with a log link to analyze feeding duration, conditional on feeding occurring (> 0 min). Food‐provisioning frequency was analyzed using a Conway–Maxwell–Poisson GLMM with a log link because preliminary Poisson models showed underdispersion. Sex and year were included as fixed effects in these parental‐care models, and nest‐day identity was included as a random intercept to account for paired male and female observations collected on the same nest‐day.

We analyzed self‐feeding and store‐food use as a binary response using a binomial GLMM, with chick age and year as fixed effects and nest‐day identity as a random intercept in previous and breeding attempt as a random intercept in the latter. We used a Poisson GLMM to analyze chick aggression frequency during feeding, with chick age and year as fixed effects and breeding attempt included as a random intercept. All models were fitted using the *glmmTMB* package in R (Magnusson et al. 2017).

To evaluate whether chick development and environmental conditions influenced parental care during chick rearing, we developed separate biologically motivated candidate models by adding one predictor at a time (chick age, daily temperature, or daily precipitation) to baseline models containing sex and year. We used the same candidate‐model approach for stored‐food use, with year included in the baseline model. Continuous predictors were standardized before analysis; candidate models were ranked using Akaike's Information Criterion corrected for small sample size (AICc), and inference was based on the top‐ranked model. We evaluated model assumptions using simulated residual diagnostics in the *DHARMa* package, including tests of residual uniformity, dispersion, and outlier. Estimated marginal means and pairwise comparisons were calculated using the *emmeans* package where appropriate.

Food types delivered to nests were identified by appearance, and photographs of some ambiguous items were taken for later confirmation of identification with expert assistance. We distinguished mammalian diets by reddish muscles and organ parts, whereas birds were characterized by light‐colored muscles, feathers, and other organs, including talons (Zhang et al. 2017).

## 3 Results

### 3.1 Breeding Phenology

Overall, Egyptian Vultures spent an average of 18.5 weeks on breeding activities, starting from nest‐building to chick fledging. Their breeding phenology and breeding season length varied between pairs and breeding seasons. Some pairs began nest repairing in late February (22.64%, *n* = 12), while most (75.47%, *n* = 40) of the nest‐repairing activities were recorded in March, with an average of 7 days (range 5–9, *n* = 53) spent on nest repairing. Only one pair started in early April (1.89%, *n* = 1). Following nest completion, adults were observed adopting an incubation‐like posture before the first egg was laid. This pre‐laying incubation‐like behavior occurred for an average of 4 days (range 2–6, *n* = 53). The first egg was laid in March in 47 breeding attempts (88.68%), and in April in 6 (11.32%). Following egg laying, true incubation lasted an average of 42 days (range 40–45, *n* = 53).

Likewise, most chick rearing started in April (67.92%, *n* = 36), while some started late in May (32.08%, *n* = 17), resulting in an average of 79 days (range 76–81, *n* = 37) for chick rearing. All successful pairs (*n* = 37) fledged their nestlings in July, with 75.67% pairs (*n* = 28) fledging before mid‐July. However, 30.18% (*n* = 16) of nests failed within the first 2 weeks of hatching.

### 3.2 Breeding Behavior

#### 3.2.1 Copulation and Nest Defense

In our study, we recorded a total of 151 copulation events in Egyptian Vultures from 14 territories, representing 20 breeding attempts. Mean copulation duration was 10.58 ± 4.28 s, with a median of 10 s and a range of 4–21 s. Copulations occurred most frequently on cliffs (*n* = 75, 49.7%), followed by trees (*n* = 68, 45.0%), and nests (*n* = 8, 5.3%). All the cliff copulations were on exposed cliff perches.

Copulation duration differed significantly among substrates (Kruskal–Wallis's χ^2^ = 92.62, df = 2, *p* < 0.001). Copulation lasted longer on cliffs (average 14 s.; range 6–21), followed by nests (average 8 s.; range 8–9) and trees (average 7 s.; range 4–11) (Figure 2). Pairwise Wilcoxon tests with Benjamini‐Hochberg adjustment indicated significant differences between cliffs and trees and between cliffs and nests (both adjusted *p* < 0.001). The difference between nests and trees was smaller (adjusted *p* = 0.042).

**FIGURE 2:**
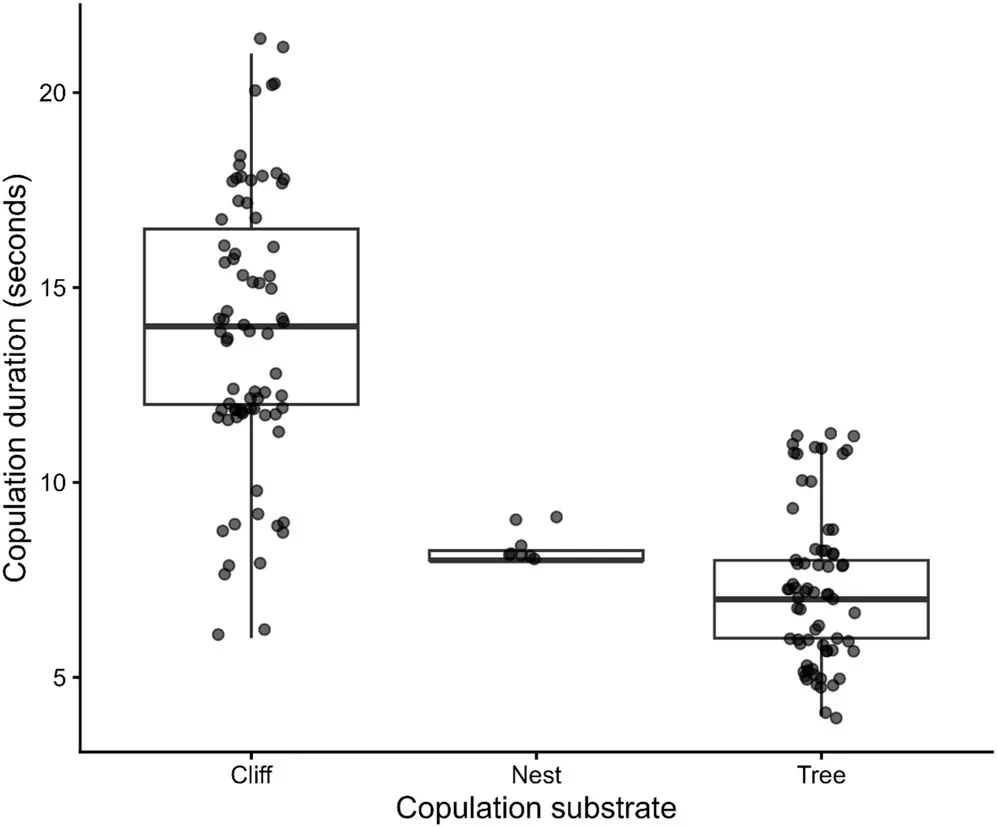
Copulation duration (seconds) of Egyptian Vultures across three copulation substrates (cliff, nest, and tree). Boxplots show the median (horizontal line), interquartile range (box), and spread of the data, with individual copulation events shown as points.

Nest defense began soon after nest building and increased after egg‐laying. Across all observation days, parents defended their nests 89 times. These include 21 cases (male = 12, female = 9) during nest‐building, 49 cases (male = 22, female = 27) during incubation, and 19 occasions (male = 8, female = 11) during chick‐rearing. Vultures defended nests either by producing hoarse warning hisses or by chasing intruders (Jungle Crows *Corvus macrorhynchos* , Common Mynas *Acridotheres tristis* , Black Kites *Milvus migrans* , Rhesus Monkeys *Macaca mulatta* , Yellow‐throated Martins *Martes falvigula*, and trespassing Egyptian Vultures). Intruding Egyptian Vultures were mostly chased by females (*n* = 27), and other intruders were chased highly by males.

#### 3.2.2 Nesting Materials and Nest Building

Between late February and early April, both members of each pair visited the nesting sites in the early morning, carrying nesting materials in their beaks. They delivered 10 different types of nesting materials of 10 different colors and placed them randomly across the entire platform, which were later managed by both members of each pair. Most nesting materials were of natural origin (68.75%), whereas anthropogenic materials accounted for 31.24% of all materials recorded. Twigs were the most frequently used nesting material (44.10%), followed by cloth pieces (27.71%), mud (8.03%), cotton (6.75%), dung (3.86%), bark (2.89%), paper (2.86%), grass (1.61%), fern (1.29%), and other materials (0.88%). Brown was the predominant color of nesting material (33.41%), followed by black (16.14%), golden (12.93%), red (11.57%), white (6.91%), ashy (5.06%), green (4.35%), blue (3.29%), yellow (3.13%), and multicolored materials (3.21%). Diversity indices (Shannon diversity, Simpson diversity) and richness of nesting material types and colors were 1.86, 0.80; 10, and 1.96, 0.82; 9, respectively.

On average, males delivered nesting materials 41.05 times (range = 19–67) per nest, while females delivered 21.2 times (range = 4–28). GLMM further showed that males delivered nest materials significantly more frequently than females, approximately 1.48 times more per observation day (Table 1). In contrast, females managed the delivered nesting materials in nests 1.64 times more frequently than males (Table 1). Material delivery and material management frequencies did not differ among years.

**TABLE 1:** Summary of the generalized linear mixed‐effects model examining the effects of sex and year on nesting materials delivery and nesting material management. The intercept represents males and year 2022. A significant negative coefficient for females indicates a lower response relative to males. Significant code: ***p < 0.001.

Fixed effects	Estimate (β)	Standard Error	*z*‐value	Pr(>|z|)
*Nest material delivery*				
Intercept (male, 2022)	1.95	0.11	18.33	< 0.001***
Sex (Female)	−0.39	0.05	−6.81	< 0.001***
Year 2023	−0.12	0.16	−0.72	0.47
Year 2024	−0.04	0.14	−0.29	0.77
*Nest material management*				
Intercept (male, 2022)	1.08	0.09	12.22	< 0.001***
Sex (Female)	0.49	0.09	5.51	< 0.001***
Year 2023	−0.001	0.12	−0.01	0.99
Year 2024	0.07	0.10	0.71	0.48

We also found strong association between the sex delivering nesting materials and the sex managing it afterwards (Pearson's χ^2^ = 287.49, simulated *p* < 0.001; Fishers exact test, *p* < 0.001). Of 521 materials delivered by males, 315 (60.5%) were managed by males and 206 (39.5%) by females. In contrast, of 346 materials delivered by females, 334 (96.5%) were managed by females and only 12 (3.5%) by males. Thus, females almost always managed the materials they delivered, whereas males managed their own materials less consistently and females managed a substantial proportion of material delivered by males.

Males and females did not differ in the origin of nesting materials (anthropogenic vs. natural) delivered (Pearson's χ^2^ = 0.036, simulated *p* = 0.895; Fisher's exact test, *p* = 0.897). However, males and females differed significantly on the composition (Pearson's χ^2^ = 97.67, simulated *p* < 0.001) and color (Pearson's χ^2^ = 137.18, simulated *p* < 0.001) of nesting materials. Males delivered a greater proportion of twigs, dung, paper, and bark and proportionally more golden, green, blue, and yellow materials than females, whereas females delivered relatively more cotton and fern and proportionally more white, black, ashy, and multicolored materials (Figure 3). Cloth was the second most frequently delivered material, and brown was the most common color delivered by both sexes.

**FIGURE 3:**
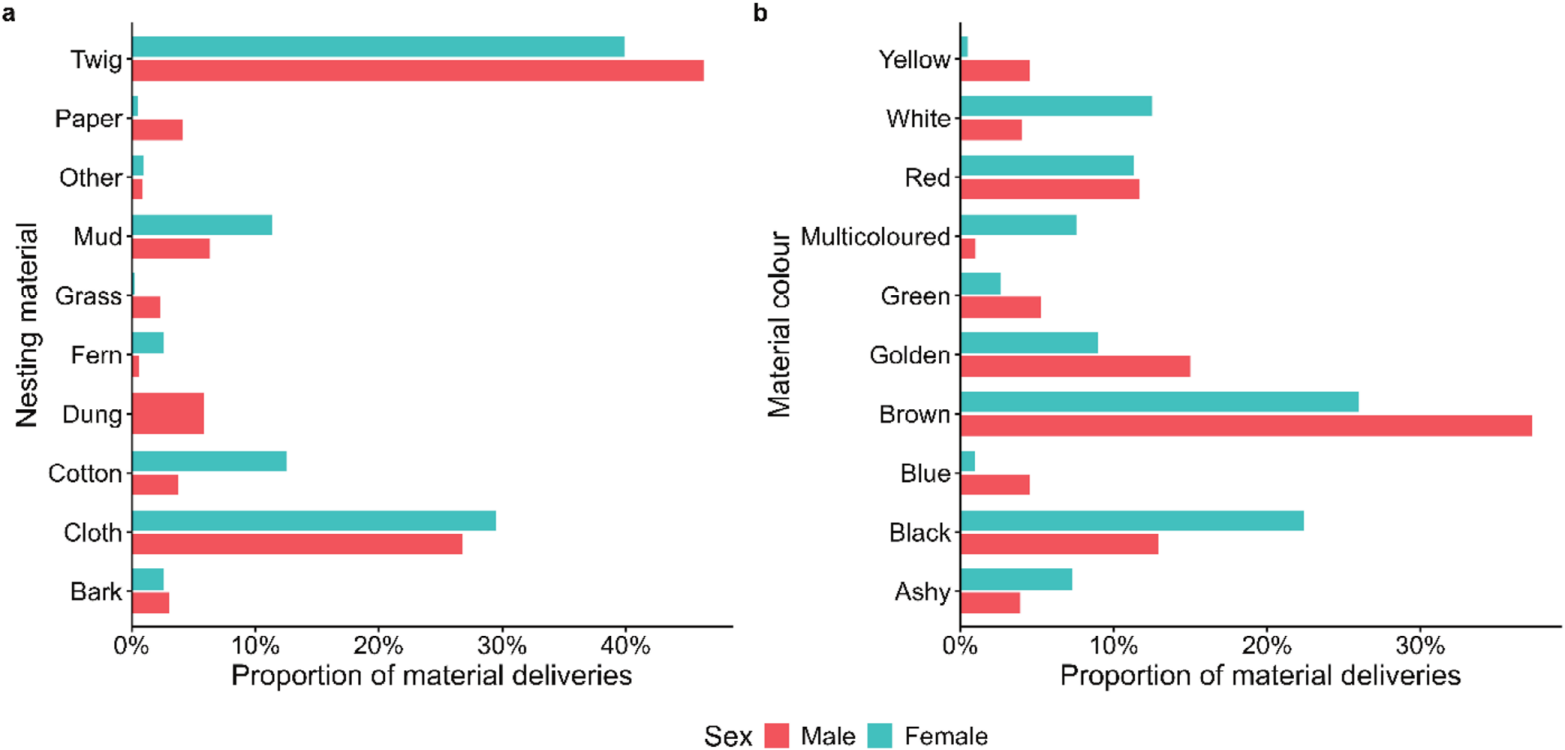
Sex‐specific variation in nesting material delivery by Egyptian Vultures during nest‐building stage. X‐axes show the proportion of material deliveries by males (orange) and females (cyan), Y‐axes show (a) nesting material type and (b) material color. Bars represent the proportion of all material deliveries recorded for each sex within each material or color category.

We observed nest‐building activity only during the early morning (7–9 am) and declined steadily throughout the afternoon.

### 3.3 Incubation

Across 18 breeding attempts across 14 territories, we conducted 1080 h of incubation observations, with each breeding attempt monitored for 5 days. Both sexes contributed substantially to incubation, although females accounted for a slightly greater proportion of the total incubation time than males (53.52% vs. 46.47%). The difference in daily incubation duration between the sexes was statistically significant (*z* = 3.46, *p* < 0.001), indicating greater daily incubation effort by females (Figure 4). The nest was unattended for only 0.006% (6.31 h) of the total observed period, with several short periods averaging 1.87 min (range 1–3 min). Because total attendance of incubating vultures per day showed almost no variation, relationships between overall daily attendance and age of eggs, sunlight duration, temperature, or precipitation were not analyzed.

**FIGURE 4:**
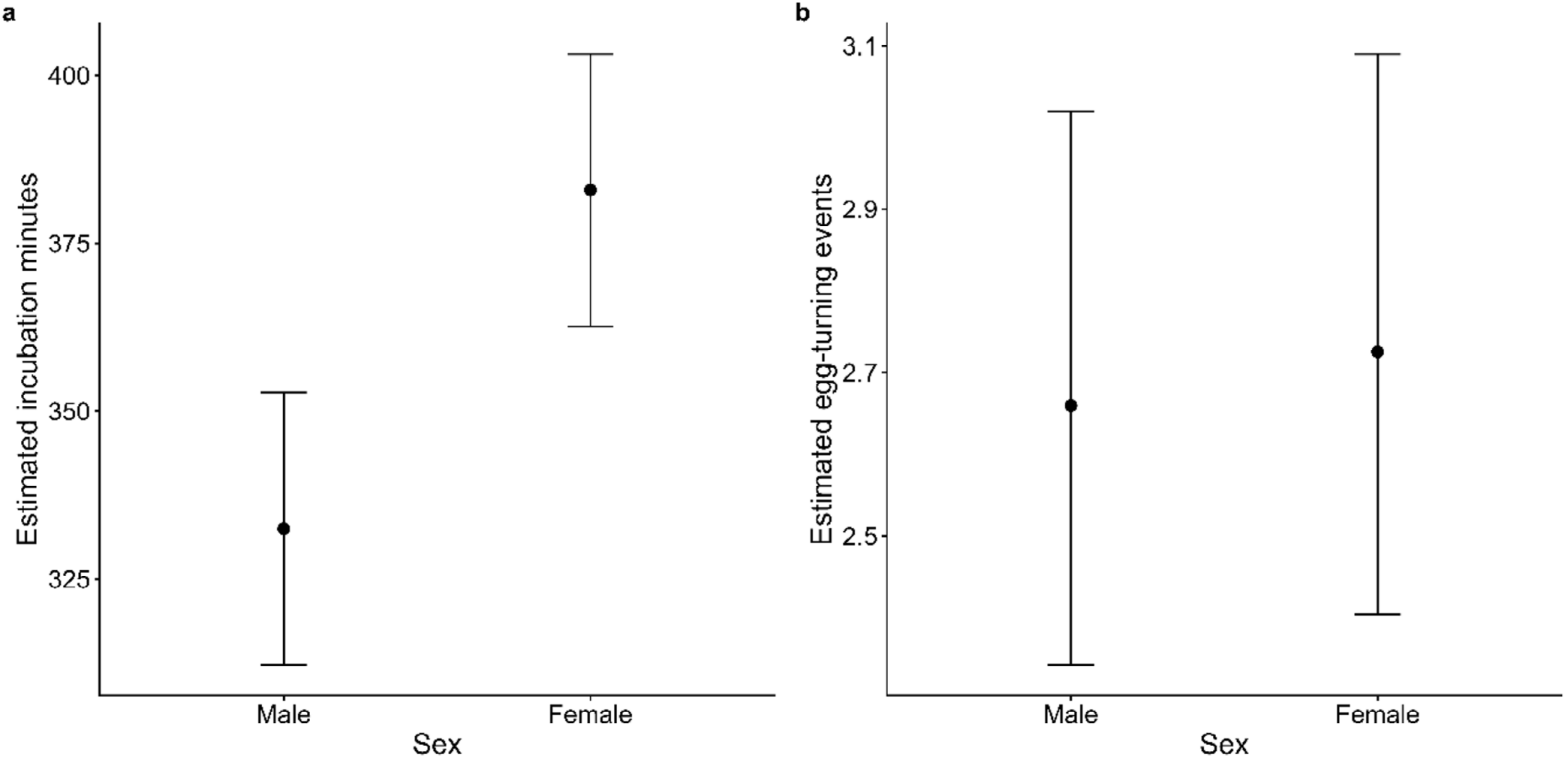
Sex‐specific differences in daily incubation effort and egg‐turning behavior of Egyptian Vultures. (a) Estimated daily incubation duration (minutes per nest per day) and (b) estimated number of egg‐turning events for males and females. Points represent the estimated means, and error bars represent 95% confidence intervals estimated from the model.

Incubation bout duration did not differ between males and females (β = 0.03 ± 0.05 SE, *z* = 0.59, *p* = 0.55). Among the candidate models to evaluate effects on incubation bout duration, the model including sunlight duration with baseline model (sex and year) received overwhelming support, whereas models including precipitation, temperature, days to hatch, or only sex received substantially less support. Incubation bout became significantly shorter as sunlight duration increased. In contrast, incubation bout was not associated with age of eggs, air temperature, sex, or year (Table 2).

**TABLE 2:** Model selection and parameter estimate for factors influencing incubation bout duration in Egyptian Vultures. Candidate generalized linear models were ranked using Akaike's Information Criterion corrected for small sample size (AICc). Models evaluated the effects of sunlight duration, precipitation, temperature, and age of eggs relative to a baseline model including sex and year. The lower section presents coefficient estimates from the best‐supported model (sunlight duration). ΔAICc is the difference in AICc relative to the top‐ranked model, and Akaike weight represents the relative support for each candidate model. Significant code: ***p < 0.001.

Model	Number of estimated parameters	AICc	ΔAICc	Akaike weight
Sunlight duration	6	7215.3	0.00	1
Precipitation	6	7257.0	41.63	< 0.001
Baseline (sex + year)	5	7263.6	48.24	< 0.001
Temperature	6	7265.3	49.95	< 0.001
Age of eggs	6	7265.4	50.07	< 0.001

Predictor	Estimate	Standard error	*z*	Pr(>|*z*|)
Parameter estimates for the top‐ranked model (sunlight duration)				
Intercept (male, 2022)	4.51	0.04	105.44	< 0.001***
Female	0.03	0.05	0.74	0.46
Sunlight duration	−0.19	0.02	−8.36	< 0.001***
Year (2023)	0.02	0.05	0.33	0.75

In total, incubation change‐over occurred 415 times, with an average of 4.5 per day (range 2–8; *n* = 90). Changeover probability differed significantly according to the sex performing the initial bout. The estimated probability of a changeover following a male bout was 0.78 (95% CI: 0.73–0.83), compared with 0.65 (95% CI: 0.59–0.71) following a female bout. The odds of a changeover were 1.92 times higher following male bouts than female bouts (*z* = 3.49, *p* < 0.001). Changeover probability was also lower in 2023 than in 2022 (β = −0.52 ± 0.19 SE, *z* = −2.61, *p* = 0.009). Age of eggs and sunlight duration were not associated with changeover probability (*p* > 0.05). While incubating the eggs, the parents changed their position by adjusting their direction 478 times, mostly facing north (38.08%), followed by south (31.62%), west (17.35%), and east (12.94%).

Eighty percent of nests contained a single egg, based on direct observations of nest contents when adults moved away from the clutch during parental changeovers and egg‐turning events. Throughout the incubation period, both parents regularly turned the eggs with their claws, totaling 490 turns; both males (242) and females (248) exhibited similar egg‐turning rates (Figure 4). A GLMM showed that egg turning frequency did not differ significantly with age of eggs, sunlight duration, precipitation, or temperature (*p* > 0.05). Individuals turned eggs more frequently in the morning compared to the afternoon.

Incubating parents maintained the nest rim by resetting the materials and scarping below the eggs to make them soft and fluffy; bill‐dig behavior. The nest repair occurred mostly during the morning hours (7 am to 11 am). Nest repair frequency differed significantly between the sexes, with males repairing 1.39 times more than females (*z* = 2.80, *p* = 0.005). However, repair frequency did not differ significantly with age of eggs (*β* = −0.08 ± 0.06 SE, *z* = −1.33, *p* = 0.19), between years (*β* = 0.23 ± 0.13 SE, *z* = 1.79, *p* = 0.074). Similarly, nest‐repairing materials delivery rates did not differ significantly between the sexes (*p* = 0.11) and years (*p* = 0.61).

### 3.4 Chick‐Rearing

We monitored Egyptian Vulture nests at different stages of the nestling period for 90 days, covering 5 observation days during 1–20, 15 days during 21–40, and 70 days during 40–66 post‐hatching days. Across all observation days, adults spent most of their time attending/guarding the nest (74.86%), followed by feeding chick (17.17%) and food provisioning (7.97%). The maximum time for total nest attendance by vultures increased as chicks became older (*β* = 0.67 ± 0.24 SE, *z* = 2.81, *p* = 0.005), with females attending the nests more significantly than males (*β* = 2.49 ± 0.65 SE, *z* = 3.82, *p* < 0.001) (Figure 5). Neither year nor precipitation significantly affected attendance probability (both *p* > 0.5). Females also remained at the nest significantly longer than males (*β* = 0.32 ± 0.11 SE, *z* = 2.79, *p* = 0.005). Attendance duration was not influenced by chick age, precipitation, or temperature (all *p* > 0.38). Likewise, attendance duration did not differ between 2022 and 2023, although attendance bouts were longer in 2024 than in 2022 (*β* = 0.29 ± 0.14 SE, *z* = 2.07, *p* = 0.039).

**FIGURE 5:**
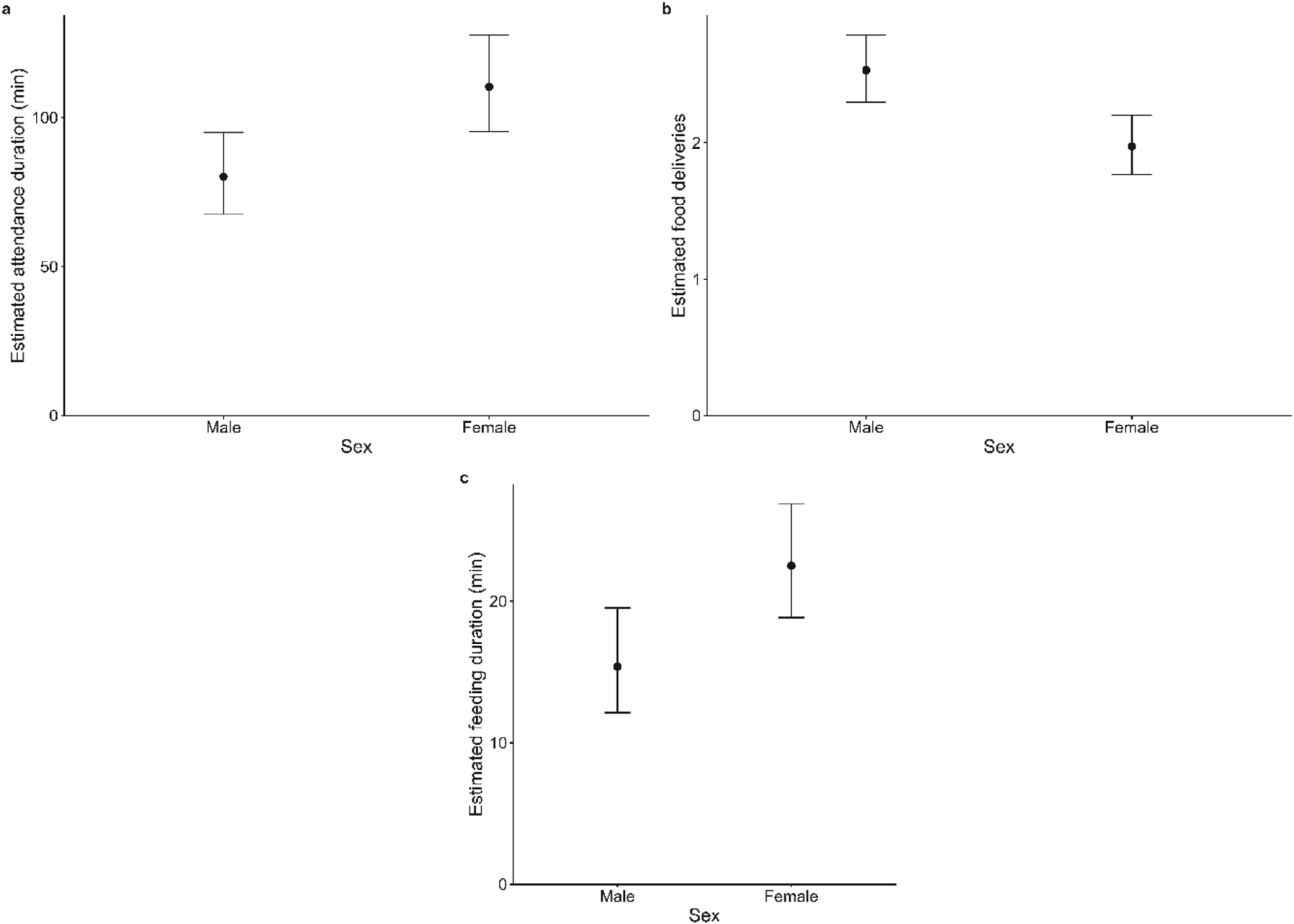
Sex‐specific variation in nest attendance, food provisioning, and chick‐feeding duration of Egyptian Vultures during the nestling/chick‐rearing period. (a) Estimated nest attendance duration (min), (b) estimated food provisioning for males and females per nest per day, and (c) estimated feeding duration (min) for males and females. Points represent model‐estimated means, and error bars represent 95% confidence intervals.

Food provisioning mostly occurred during mornings (8–12 am, 56.98%) and evenings (4–6 pm, 20.95%), followed by other parts of the day (< 8 am, 8.88%; 12–4 pm, 13.17%). The food provisioning rate was best explained by the model containing chick age with the baseline model (sex and year) (AICc weight = 1.00). The top‐ranked model suggested that the food‐provisioning rate increased significantly with chick age, males deliver food more frequently than females (Figure 6), and provisioning was higher in the year 2023 (Table 3). Both parents delivered food to the nestling via their beaks, consisting of poultry (65%), mammalian meat (15.77%), and unidentified meat (19.23%). The chi‐squared test indicated no significant differences in the types of food brought by males and females (χ^2^ = 5.96, df = 9, *p* = 0.74). They brought food at different intervals, averaging 5.37 occasions per day (range 2–12), and it was either fed immediately or reserved on the nest platform. For nests with two chicks, parents brought food on average 8 times per day (range: 4–10). However, the food delivery rate differed significantly between the sexes; males made an estimated 1.28 times more than that of females (z = 3.75, *p* < 0.001) (Figure 5). Provisioning rate did not differ significantly among years (*p* > 0.05).

**FIGURE 6:**
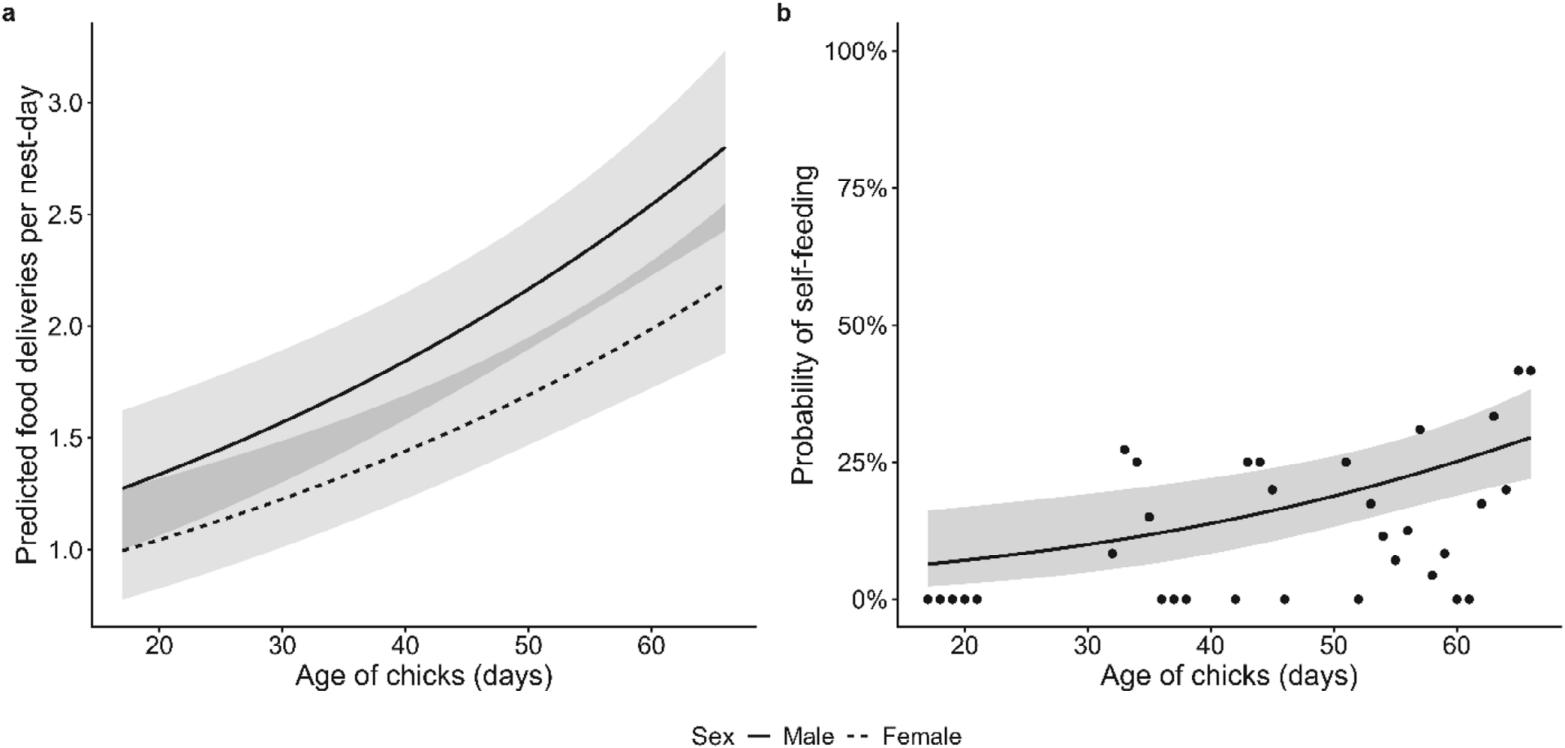
Changes in chick provisioning behavior of Egyptian Vultures with chick age. (a) Predicted number of food deliveries per nest per day by males (solid line) and females (dashed line) in relation to chick age. (b) Predicted probability of self‐feeding by chicks in relation to chick age; points represent observed proportions. Shaded areas represent 95% confidence intervals around model predictions.

**TABLE 3:** Model selection and parameter estimate for factors influencing food provisioning in Egyptian Vultures. Candidate generalized linear models with a Conway–Maxwell–Poisson error distribution and log link were ranked using Akaike's Information Criterion corrected for small sample size (AICc). Models evaluated the effects of chick age, daily precipitation, and daily temperature relative to a baseline model including sex and year. The lower section presents coefficient estimates from the best‐supported model (chick age). ΔAICc is the difference in AICc relative to the top‐ranked model, and Akaike weight represents the relative support for each candidate model. Significant code: ***p < 0.001.

Model	Number of estimated parameters	AICc	ΔAICc	Akaike weight
Sex + Year + Chick age	7	542.7	0.00	1
Sex + Year (baseline)	6	572.3	29.63	< 0.001
Sex + Year + Precipitation	7	574.0	31.27	< 0.001
Sex + Year + Temperature	7	574.2	31.48	< 0.001

Predictor	Estimate (β)	Standard error	*z*	Pr(>|*z*|)
Parameter estimates for the top‐ranked model (chick age)				
Intercept (male, 2022)	0.76	0.07	11.16	< 0.001***
Female vs. Male	−0.25	0.07	−3.80	< 0.001***
Chick age	0.22	0.04	5.86	< 0.001***
Year 2023	0.27	0.08	3.36	< 0.001***
Year 2024	0.21	0.09	2.38	0.02

Females fed nestling on all monitored nest‐days (90/90), whereas males fed on 53 of 90 monitored nest‐days. Because feeding occurrence for females exhibited complete separation, statistical modeling of feeding occurrence was not performed. Similarly, females spent significantly longer feeding nestlings than males (β = 0.38 ± 0.11 SE, *z* = 3.59, *p* < 0.001) (Figure 5) but did not differ between years (all *p* > 0.53). Variation in duration of feeding was strongly supported by the model including precipitation (AICc weight = 1.00), whereas models including chick age, temperature, or no environmental predictor received essentially no support (AICc ≥ 21.96). The model suggests feeding duration declined significantly with increasing precipitation (*β* = −0.47 ± 0.07 SE, *z* = −6.60, *p* < 0.001). Daily temperature did not influence feeding duration (*β* = −0.05 ± 0.08 SE, *p* = 0.54). Although chick age was negatively associated with feeding duration when analyzed alone (*β* = −0.31 ± 0.08 SE, *p* < 0.001), this model had substantially less support than the precipitation model (ΔAICc = 21.96), indicating that precipitation explained variation in feeding duration more effectively.

The probability of self‐feeding increased significantly with chick age (*β* = 0.46 ± 0.15 SE, *z* = 3.15, *p* = 0.002) (Figure 6). In contrast, self‐feeding probability did not differ between 2022 and 2023 (*p* = 0.68) but was significantly lower in 2024 (*β* = −1.79 ± 0.44 SE, *z* = −4.07, *p* < 0.001). The use of stored food for feeding was better explained by temperature (AICc = 101.6, model weight = 0.48) and chick age (ΔAICc = 0.87, model weight = 0.31) models, suggesting that the probability of stored‐food use increased significantly with temperature (*β* = 1.18 ± 0.56 SE, *z* = 2.13, *p* = 0.033) and chick age (*β* = 1.36 ± 0.76 SE, *z* = 1.80, *p* = 0.072), although the latter was not statistically significant. Chick aggression during feeding also increased significantly with chick age (*β* = 0.46 ± 0.12 SE, *z* = 3.98, *p* < 0.001) (Figure 7), whereas year had no detectable effect (*p* > 0.46). However, we didn't detect evidence of interaction between nestlings and cases of siblicide in our study.

**FIGURE 7:**
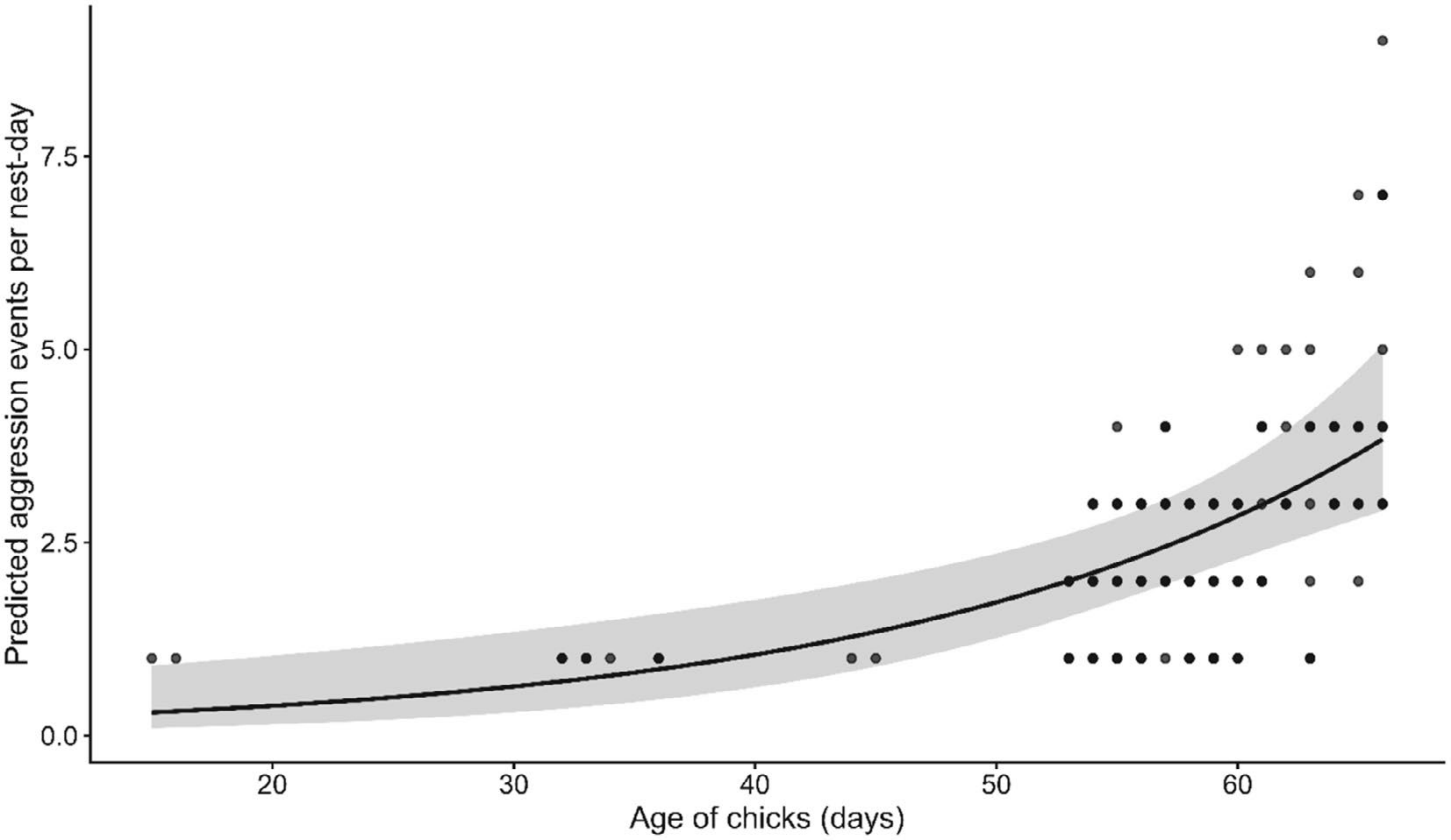
Relationship between chick age (days) and aggression frequency in Egyptian vultures during the nestling period. The solid line represents the model‐predicted number of aggression events per nest per day in relation to chick age, with the shaded area indicating the 95% confidence intervals. Points represent observed aggression frequencies.

## 4 Discussion

Our study showed that Egyptian Vultures exhibit biparental care throughout chick development. Cooperative parental care likely reduced the cost of investing maximum time in offspring (Morant Etxebarria et al. 2019) and searching for spatially and temporally unpredictable food resources (Margalida and Bertran 2000) for a single adult. However, we observed behavioral asymmetries in parental investment across specific activities and breeding stages.

Egyptian Vultures initiated their breeding cycle with synchronized nest building and courtship, which may reflect coordination with the period of sperm viability and fertile female period (Donazar et al. 1994). Egyptian Vultures showed considerable variation among nests and breeding seasons in the timing of breeding activities, while incubation and chick‐rearing durations were generally within ranges previously reported for the species (Donazar and Ceballos 1989; Şen 2012; Morant Etxebarria et al. 2019; Kumar et al. 2020). Although some aspects of breeding chronology differed from observations reported by Kumar et al. (2020) in India, their study was based on a single nest, whereas our study included multiple nests and multiple breeding attempts. Larger comparative studies across the species' range are needed to determine the extent of geographical variation in breeding phenology.

Local environmental conditions may nevertheless contribute to variation in breeding chronology. Most nests in our study occurred in human‐dominated landscapes, where disturbance could influence the timing and expression of breeding activities. Egyptian Vultures are highly cautious when approaching their nests and may delay entry until surrounding disturbance subsides (Zuberogoitia et al. 2008); consequently, breeding pairs may reduce conspicuous nest‐building activities under disturbed conditions (Morant et al. 2018). Although we observed no obvious behavioral responses to our presence, subtle observer effects cannot be excluded. Food availability and seasonal weather conditions may influence parental foraging and chick development, particularly because predictable food sources in the study area have declined following landfill closure (Baral, Neupane, and Subedi 2026) and the breeding season overlaps with substantial variation in temperature and rainfall (Paudel 2020). Thus, rather than indicating fixed geographical differences in breeding phenology, the variation observed among nests and years may reflect the flexibility of breeding pairs in response to local conditions.

Egyptian Vultures delivered nesting materials exclusively during the morning and used a more diverse range of materials, consistent with previous observations (Mishra et al. 2017; Kumar et al. 2020). Morning delivery may reduce exposure to human disturbance (Morant Etxebarria et al. 2019) while allowing birds to prioritize nest‐building activities before stronger thermals facilitate longer‐distance foraging flights (Duriez et al. 2014). Material use likely reflected local availability (James Reynolds et al. 2019), with cloth primarily used for nest lining and twigs contributing nest structure; the predominance of brown and black materials may also provide camouflage against the nesting substrate (Bailey et al. 2015). Males delivered nesting materials more frequently than females, consistent with Yordanov et al. (2022), and also differed in types of materials transported. Males frequently delivered heavier structural materials such as twigs, whereas females predominantly delivered lighter materials such as cotton and fern, suggesting a functional division of labor in which males contribute more to nest structure and females to nest lining and cup formation. This division may reduce the energetic costs of material transport for females during the energetically demanding period of egg production (Chastel et al. 1995; Margalida and Bertran 2000). Observed differences in material color likely reflected differences in material type rather than active color selection, as cotton was typically white and golden coloration occurred exclusively in twigs.

Following the onset of incubation, Egyptian Vultures exhibited biparental care of the clutch and young nestlings, with nests unattended during parental changeovers, nest repair, position adjustments, and egg turning. Such high nest attendance likely facilitates egg thermoregulation and protection from insolation and predation (AlRashidi et al. 2010; Deeming and Reynolds 2015; Morant Etxebarria et al. 2019). Females incubated more than males, whereas males performed slightly more nest repair, suggesting complementary parental roles that may reduce the costs of reproduction for females. Regular changeovers further allow parents to share energetic demands of incubation while maintaining opportunities to forage, particularly when food is spatially and temporally unpredictable (Deygout et al. 2010; López‐López et al. 2014; Marasco and Spencer 2015; Morant Etxebarria et al. 2019). Because adults did not provision their incubating partner during changeovers, relief frequency may depend on the foraging success of non‐incubating parents (Margalida and Bertran 2000; Rollack et al. 2013) and local food availability (Monsarrat et al. 2013; Cortés‐Avizanda et al. 2016). Incubation bouts became shorter with increasing sunlight exposure, consistent with increased changeover rates under warmer conditions reported by Morant Etxebarria et al. (2019), potentially because favorable thermal conditions improve foraging opportunities (Dodge et al. 2014). Changeover rates were also lower in 2023 than in 2022, coinciding with irregular rainfall during incubation. Rainfall may restrict soaring opportunities and consequently reduce the frequency with which non‐incubating parents can forage and relieve their partners (Duriez et al. 2014). In contrast, egg‐turning frequency was unaffected by parental sex, egg age, or environmental conditions, possibly because most observations occurred late in incubation, when embryos were well developed and turning requirements may decline as hatching approaches (Tona et al. 2003).

Both parents contributed to chick rearing, but their roles differed. Females spent more time attending nests and feeding nestlings, whereas males delivered food more frequently, indicating a division of parental investment. High attendance likely provides young chicks with protection from thermal stress and predation (Brown 2002; Margalida et al. 2007; Morant Etxebarria et al. 2019). Longer nest attendance in 2024 likely reflects the younger age of chicks monitored that year.

Poultry remains, followed by mammalian remains, dominated chick provisioning, consistent with previous dietary observation in Kaski District (Baral, Neupane, Gurung, et al. 2026). Food was delivered primarily during the morning, potentially reflecting greater food availability from landfills, poultry farms, and slaughterhouses during this period (Baral, Neupane, Gurung, et al. 2026). Provisioning rates increased with chick age, consistent with increasing energetic and nutritional demands of growing nestlings (Newton 2010; Morant Etxebarria et al. 2019), whereas feeding duration decreased with increasing precipitation, potentially because rainfall reduces foraging opportunities and soaring efficiency, resulting in less returns of adults to nests with food (Duriez et al. 2014). Similarly, higher chick mortality in 2023 also coincided with irregular rainfall during incubation and early chick‐rearing periods. Together with lower parental changeover rates and reduced feeding duration under wetter conditions, this pattern suggests that adverse weather may constrain parental care and food availability during early development. However, because causes of mortality were not directly assessed, this explanation remains speculative. The higher provisioning rates observed in 2023 should also be interpreted cautiously, as early nest failures meant that chick‐rearing observations were disproportionately obtained from nests with older chicks, which naturally received higher provisioning rates.

The probability of using stored food increased with the temperature, potentially reflecting a trade‐off between improved soaring efficiency and thermoregulatory constraints during warmer periods. Higher temperatures can strengthen thermal updrafts, allowing vultures to travel greater distances at relatively low energetic cost and potentially forage over larger areas, which may reduce the frequency of returns to the nest and direct food provisioning. Alternatively, during periods of high temperature, vultures may reduce activity and increase resting behaviors to minimize heat stress. Under either scenario, greater reliance on food previously stored at the nest may maintain chick nourishment when fresh provisioning visits are less frequent.

The self‐feeding by nestling increased with the chick age, reflecting the increasing ability of nestling to handle food independently (Bassi et al. 2017). Aggression toward parents also increased with chick age, consistent with increasing offspring‐parent conflict as nestling energetic demands increase (Newton 2010; Morant Etxebarria et al. 2019; Morandini et al. 2021). Aggression was directed more frequently toward females, potentially because females were more directly involved in feeding nestlings.

Overall, our findings suggest that the breeding onset of Egyptian Vultures varies across nests and breeding seasons. They invested asymmetric biparental effort at varying levels across the stages of their breeding cycle. Both parents showed similar levels of parental care in incubation and egg‐turning. Males contributed more to nesting material delivery and food delivery, while females were more active in nest construction and nestling feeding. Together, these findings reveal a flexible division of parental roles in which both members of the breeding pair contribute to offspring care but allocate their effort differently across breeding stages and activities. Such complementary parental investment may be particularly important for meeting the prolonged reproductive demands of long‐lived species such as the Egyptian Vulture.

## Author Contributions

**Milan Baral:** conceptualization (lead), data curation (lead), formal analysis (lead), funding acquisition (lead), investigation (lead), methodology (lead), project administration (lead), writing – original draft (lead), writing – review and editing (lead). **Anisha Neupane:** conceptualization (supporting), data curation (supporting), formal analysis (supporting), funding acquisition (supporting), investigation (supporting), writing – review and editing (supporting). **Sandesh Gurung:** conceptualization (supporting), funding acquisition (supporting), methodology (supporting), supervision (supporting), writing – review and editing (supporting). **Tulsi Ram Subedi:** conceptualization (supporting), funding acquisition (supporting), methodology (supporting), supervision (supporting), writing – review and editing (supporting).

## Funding

The Rufford Foundation UK funded the research project through the Rufford Small Grants. Grant ID: 39814‐1.

## Conflicts of Interest

The authors declare no conflicts of interest.

## Supporting information


**Data S1:** Supporting Information.

## Data Availability

All the required data are uploaded as Supporting Information.

## References

[ece374406-bib-0001] AlRashidi, M. , A. Kosztolányi , C. Küpper , I. C. Cuthill , S. Javed , and T. Székely . 2010. “The Influence of a Hot Environment on Parental Cooperation of a Ground‐Nesting Shorebird, the Kentish Plover *Charadrius alexandrinus* .” Frontiers in Zoology 7: 1. 10.1186/1742-9994-7-1 PMC2819062 20148101

[ece374406-bib-0002] Bailey, I. E. , F. Muth , K. Morgan , S. L. Meddle , and S. D. Healy . 2015. “Birds Build Camouflaged Nests.” Auk: Ornithological Advances 132: 11–15.

[ece374406-bib-0003] Baral, M. , A. Neupane , S. Gurung , T. R. Subedi , and R. Koirala . 2026. “Age‐Class Differences in the Feeding Ecology of Egyptian Vultures ( *Neophron percnopterus* ) at Pokhara Landfill in Nepal.” Journal of Raptor Research 60: 1–14.

[ece374406-bib-0004] Baral, M. , A. Neupane , and T. R. Subedi . 2026. “Roost Habitat Selection and Population Status of Globally Endangered Egyptian Vultures ( *Neophron percnopterus* ) in the Kaski District of Central‐West Nepal.” Journal of Raptor Research 61: 1–15.

[ece374406-bib-0005] Bassi, E. , P. Trotti , M. Brambilla , et al. 2017. “Parental Investment in Two Large Raptors Breeding in a High Prey Density Area.” Journal of Ornithology 158: 549–559.

[ece374406-bib-0006] Brooker, R. M. , W. E. Feeney , J. R. White , R. P. Manassa , J. L. Johansen , and D. L. Dixson . 2016. “Using Insights From Animal Behaviour and Behavioural Ecology to Inform Marine Conservation Initiatives.” Animal Behaviour 120: 211–221. 10.1016/j.anbehav.2016.03.012 PMC5665575 29104297

[ece374406-bib-0007] Brown, C. 2002. “Avian Incubation: Behaviour, Environment, and Evolution, Edited by DC Deeming: Book Review.” African Zoology 37: 263–264.

[ece374406-bib-0008] Bulla, M. , M. Valcu , A. M. Dokter , et al. 2016. “Unexpected Diversity in Socially Synchronized Rhythms of Shorebirds.” Nature 540: 109–113. 10.1038/nature20563 27880762

[ece374406-bib-0009] Chastel, O. , H. Weimerskirch , and P. Jouventin . 1995. “Influence of Body Condition on Reproductive Decision and Reproductive Success in the Blue Petrel.” Auk 112: 964–972.

[ece374406-bib-0010] Clark, W. S. , and J. N. Schmitt . 1998. “Aeging Egyptian Vulture.” Alula 4: 122–127.

[ece374406-bib-0011] Cortés‐Avizanda, A. , G. Blanco , T. L. DeVault , et al. 2016. “Supplementary Feeding and Endangered Avian Scavengers: Benefits, Caveats, and Controversies.” Frontiers in Ecology and the Environment 14: 191–199.

[ece374406-bib-0012] Cresswell, W. , S. Holt , J. Reid , D. Whitfield , and R. Mellanby . 2003. “Do Energetic Demands Constrain Incubation Scheduling in a Biparental Species?” Behavioral Ecology 14: 97–102.

[ece374406-bib-0013] Deeming, D. C. , and S. J. Reynolds . 2015. Nests, Eggs, and Incubation: New Ideas About Avian Reproduction. Oxford University Press.

[ece374406-bib-0014] Deygout, C. , A. Gault , O. Duriez , F. Sarrazin , and C. Bessa‐Gomes . 2010. “Impact of Food Predictability on Social Facilitation by Foraging Scavengers.” Behavioral Ecology 21: 1131–1139.

[ece374406-bib-0015] Dodge, S. , G. Bohrer , K. Bildstein , et al. 2014. “Environmental Drivers of Variability in the Movement Ecology of Turkey Vultures ( *Cathartes aura* ) in North and South America.” Philosophical Transactions of the Royal Society, B: Biological Sciences 369: 20130195. 10.1098/rstb.2013.0195 PMC3983930 24733950

[ece374406-bib-0016] Donazar, J. A. , and O. Ceballos . 1989. “ *Neophron percnopterus* in Relation to Brood Size, Hatching Order and Environmental, Factors.” Ardea 77: 2.

[ece374406-bib-0017] Donazar, J. A. , O. Ceballos , and J. L. Tella . 1994. “Copulation Behaviour in the Egyptian Vulture *Neophron percnopterus* .” Bird Study 41: 37–41.

[ece374406-bib-0018] Duriez, O. , A. Kato , C. Tromp , et al. 2014. “How Cheap Is Soaring Flight in Raptors? A Preliminary Investigation in Freely‐Flying Vultures.” PLoS One 9: e84887. 10.1371/journal.pone.0084887 PMC3893159 24454760

[ece374406-bib-0019] Eldegard, K. , V. Selås , G. A. Sonerud , C. Steel , and T. Rafoss . 2003. “The Effect of Parent Sex on Prey Deliveries to Fledgling Eurasian Sparrowhawks *Accipiter nisus* .” Ibis 145: 667–672.

[ece374406-bib-0020] Eldegard, K. , and G. A. Sonerud . 2012. “Sex Roles During Post‐Fledging Care in Birds: Female Tengmalm's Owls Contribute Little to Food Provisioning.” Journal of Ornithology 153: 385–398.

[ece374406-bib-0021] Fischer, M. , J. Brettin , S. Roessner , A. Walz , M. Fort , and O. Korup . 2022. “Rare Flood Scenarios for a Rapidly Growing High‐Mountain City: Pokhara, Nepal.” Natural Hazards and Earth System Sciences 22: 3105–3123.

[ece374406-bib-0022] Ghimire, M. , H. Chaudhary , and H. Dhakal . 2019. Birds of Pokhara Valley. Pokhara Bird Society.

[ece374406-bib-0023] Ghimire, P. , H. Dhakal , B. Sharma , M. Ghimire , M. Baral , and K. P. Bhusal . 2020. “Status of Egyptian Vulture *Neophron percnopterus* in Pokhara Valley, Nepal.” Vulture Bull 9: 1–4.

[ece374406-bib-0024] Gorelick, N. , M. Hancher , M. Dixon , S. Ilyushchenko , D. Thau , and R. Moore . 2017. “Google Earth Engine: Planetary‐Scale Geospatial Analysis for Everyone.” Remote Sensing of Environment 202: 18–27.

[ece374406-bib-0025] Gurung, S. , T. R. Subedi , R. Baral , et al. 2023. “Breeding Habitat and Factors Affecting the Cliff Selection by Egyptian Vultures in Central‐West Nepal.” Journal of Raptor Research 57: 81–91.

[ece374406-bib-0026] Hartig, F. , and M. F. Hartig . 2017. “Package ‘dharma’.” R package 531:532.

[ece374406-bib-0027] Henshaw, J. M. , L. Fromhage , and A. G. Jones . 2019. “Sex Roles and the Evolution of Parental Care Specialization.” Proceedings of the Royal Society B: Biological Sciences 286: 20191312. 10.1098/rspb.2019.1312 PMC6732396 31455191

[ece374406-bib-0028] Inskipp, C. , H. Baral , S. Phuyal , et al. 2016. The Status of Nepal's Birds: The National Red List Series. Zoological Society of London.

[ece374406-bib-0029] Jackson, A. L. , G. D. Ruxton , and D. C. Houston . 2008. “The Effect of Social Facilitation on Foraging Success in Vultures: A Modelling Study.” Biology Letters 4: 311–313. 10.1098/rsbl.2008.0038 PMC2610049 18364309

[ece374406-bib-0030] James Reynolds, S. , J. D. Ibáñez‐Álamo , P. Sumasgutner , and M. C. Mainwaring . 2019. “Urbanisation and Nest Building in Birds: A Review of Threats and Opportunities.” Journal of Ornithology 160: 841–860.

[ece374406-bib-0031] Khim Bahadur, K. C. , N. P. Koju , K. P. Bhusal , et al. 2019. “Factors Influencing the Presence of the Endangered Egyptian Vulture *Neophron percnopterus* in Rukum, Nepal.” Global Ecology and Conservation 20: e00727.

[ece374406-bib-0032] Kovařík, P. , V. Pavel , and B. Chutný . 2009. “Incubation Behaviour of the Meadow Pipit ( *Anthus pratensis* ) in an Alpine Ecosystem of Central Europe.” Journal of Ornithology 150: 549–556.

[ece374406-bib-0033] Kumar, C. , A. S. Kaleka , and S. K. Thind . 2020. “Observations on Breeding Behaviour of a Pair of Endangered Egyptian Vultures *Neophron percnopterus* (Linnaeus, 1758) Over Three Breeding Seasons in the Plains of Punjab, India.” Journal of Threatened Taxa 12: 16013–16020.

[ece374406-bib-0034] López‐López, P. , C. García‐Ripollés , and V. Urios . 2014. “Food Predictability Determines Space Use of Endangered Vultures: Implications for Management of Supplementary Feeding.” Ecological Applications 24: 938–949. 10.1890/13-2000.1 25154088

[ece374406-bib-0035] Magnusson, A. , H. Skaug , A. Nielsen , et al. 2017. “Package ‘glmmtmb’.” R Package Version 0.2.2 25.

[ece374406-bib-0036] Marasco, V. , and K. Spencer . 2015. “Improvements in Our Understanding of Behaviour During Incubation.” In Nests, Eggs and Incubation: New Ideas About Avian Incubation, edited by D. C. Deeming and S. J. Reynolds , 142–151. Oxford University Press.

[ece374406-bib-0037] Margalida, A. , J. R. Benitez , J. A. Sanchez‐Zapata , A. Enrique , R. Arenas , and J. A. Donazar . 2012. “Long‐Term Trends in Population Size and Breeding Success in the Egyptian Vulture ( *Neophron percnopterus* ) in Northern Spain.” Ibis 142, no. 2: 184–188.

[ece374406-bib-0038] Margalida, A. , and J. Bertran . 2000. “Breeding Behaviour of the Bearded Vulture *Gypaetus barbatus* : Minimal Sexual Differences in Parental Activities.” Ibis 142: 225–234.

[ece374406-bib-0040] Margalida, A. , L. M. González , R. Sánchez , J. Oria , and L. Prada . 2007. “Parental Behaviour of Spanish Imperial Eagles *Aquila adalberti* : Sexual Differences in a Moderately Dimorphic Raptor.” Bird Study 54: 112–119.

[ece374406-bib-0041] Mateo‐Tomás, P. , and P. P. Olea . 2015. “Livestock‐Driven Land Use Change to Model Species Distributions: Egyptian Vulture as a Case Study.” Ecological Indicators 57: 331–340.

[ece374406-bib-0042] McClure, C. J. 2025. “The Need to Assess the State of the World's Raptors.” In State of the World's Raptors. Peregrine Fund.

[ece374406-bib-0043] Merrick, M. J. , and J. L. Koprowski . 2017. “Should We Consider Individual Behavior Differences in Applied Wildlife Conservation Studies?” Biological Conservation 209: 34–44.

[ece374406-bib-0044] Milchev, B. , N. Spassov , and V. Popov . 2012. “Diet of the Egyptian Vulture ( *Neophron percnopterus* ) After Livestock Reduction in Eastern Bulgaria.” North‐Western Journal of Zoology 8: 315–323.

[ece374406-bib-0045] Mishra, S. , A. Kumar , and A. Kanaujia . 2017. “Nest Material Selection by Egyptian Vulture *Neophron percnopterus* .” Journal of Entomology and Zoology Studies 5: 1649–1655.

[ece374406-bib-0046] Mishra, S. , A. Kumar , and A. Kanaujia . 2018. “A Review on Subspecies of Egyptian Vulture.” Journal of New Biological Reports 7: 60–67.

[ece374406-bib-0047] Monsarrat, S. , S. Benhamou , F. Sarrazin , C. Bessa‐Gomes , W. Bouten , and O. Duriez . 2013. “How Predictability of Feeding Patches Affects Home Range and Foraging Habitat Selection in Avian Social Scavengers?” PLoS One 8: e53077. 10.1371/journal.pone.0053077 PMC3536817 23301024

[ece374406-bib-0048] Morandini, V. , J. Viñuela , J. Belliure , and M. Ferrer . 2021. “Parent–Offspring Conflict and Transition to crèche Phase in Chinstrap Penguins ( *Pygoscelis antarctica* ).” Polar Biology 44: 1961–1966.

[ece374406-bib-0049] Morant Etxebarria, J. , P. López‐López , and I. Zuberogoitia Arroyo . 2019. “Parental Investment Asymmetries of a Globally Endangered Scavenger: Unravelling the Role of Gender, Weather Conditions and Stage of the Nesting Cycle.” Bird Study 66: 329–341.

[ece374406-bib-0050] Morant, J. , J. Zabala , J. Martínez , and I. Zuberogoitia . 2018. “Out of Sight, Out of Mind? Testing the Effects of Overwinter Habitat Alterations on Breeding Territories of a Migratory Endangered Species.” Animal Conservation 21: 465–473.

[ece374406-bib-0051] Munoz‐Sabater, J. , E. Dutra , A. Agusti‐Panareda , et al. 2021. “ERA5‐Land: A State‐Of‐The‐Art Global Reanalysis Dataset for Land Applications.” Earth System Science Data 13: 4349–4383.

[ece374406-bib-0052] Newton, I. 2010. Population Ecology of Raptors. A&C Black.

[ece374406-bib-0053] Oppel, S. , V. Dobrev , V. Arkumarev , et al. 2017. “Landscape Factors Affecting Territory Occupancy and Breeding Success of Egyptian Vultures on the Balkan Peninsula.” Journal of Ornithology 158: 443–457.

[ece374406-bib-0054] Paudel, U. 2020. “Trends of Temperature and Rainfall in Pokhara.” Prithvi Academic Journal 3: 22–32.

[ece374406-bib-0055] Remeš, V. , R. P. Freckleton , J. Tökölyi , A. Liker , and T. Székely . 2015. “The Evolution of Parental Cooperation in Birds.” Proceedings of the National Academy of Sciences 112: 13603–13608. 10.1073/pnas.1512599112 PMC4640770 26483476

[ece374406-bib-0056] Renner, S. S. , and C. M. Zohner . 2018. “Climate Change and Phenological Mismatch in Trophic Interactions Among Plants, Insects, and Vertebrates.” Annual Review of Ecology, Evolution, and Systematics 49: 165–182.

[ece374406-bib-0057] Rollack, C. E. , K. Wiebe , M. J. Stoffel , and C. S. Houston . 2013. “Turkey Vulture Breeding Behavior Studied With Trail Cameras.” Journal of Raptor Research 47: 153–160.

[ece374406-bib-0058] Şen, B. 2012. Breeding Ecology of the Egyptian Vulture ( *Neophron percnopterus* ) Population Beypazarı. Middle East Technical University.

[ece374406-bib-0059] Smith, P. A. , I. Tulp , H. Schekkerman , H. G. Gilchrist , and M. R. Forbes . 2012. “Shorebird Incubation Behaviour and Its Influence on the Risk of Nest Predation.” Animal Behaviour 84: 835–842.

[ece374406-bib-0060] Subedi, T. R. , and R. DeCandido . 2015. “Population and Breeding Success of Red‐Headed Vulture *Sarcogyps calvus* and Egyptian Vulture *Neophron percnopterus* in Central West Nepal.” Vulture News 67: 21–32.

[ece374406-bib-0061] Tona, K. , O. Onagbesan , B. De Ketelaere , E. Decuypere , and V. Bruggeman . 2003. “Effects of Turning Duration During Incubation on Corticosterone and Thyroid Hormone Levels, Gas Pressures in Air Cell, Chick Quality, and Juvenile Growth.” Poultry Science 82: 1974–1979. 10.1093/ps/82.12.1974 14717556

[ece374406-bib-0062] Yordanov, E. S. , V. D. Dobrev , and A. Jambon . 2022. “Trail Cameras Reveal New Details of the Breeding Behaviour of an Endangered Vulture Species.” Page 9 *in* Proceedings of the Egyptian Vulture Conference.

[ece374406-bib-0063] Zhang, X. , C. M. Owens , and M. W. Schilling . 2017. “Meat: The Edible Flesh From Mammals Only or Does It Include Poultry, Fish, and Seafood?” Animal Frontiers 7: 12–18.

[ece374406-bib-0064] Zuberogoitia, I. , J. E. Martínez , M. Larrea , and J. Zabala . 2018. “Parental Investment of Male Peregrine Falcons During Incubation: Influence of Experience and Weather.” Journal of Ornithology 159: 275–282.

[ece374406-bib-0065] Zuberogoitia, I. , J. Zabala , J. Martínez , J. Martínez , and A. Azkona . 2008. “Effect of Human Activities on Egyptian Vulture Breeding Success.” Animal Conservation 11: 313–320.

